# Assessment of the Sensitizing Potential of Proteins in BALB/c Mice: Comparison of Three Protocols of Intraperitoneal Sensitization

**DOI:** 10.3390/nu10070903

**Published:** 2018-07-14

**Authors:** Jesús Gilberto Arámburo-Galvez, Norberto Sotelo-Cruz, Lilian Karem Flores-Mendoza, Martina Hilda Gracia-Valenzuela, Francisco Iván Rodolfo Chiquete-Elizalde, Jesús Guadalupe Espinoza-Alderete, Humberto Trejo-Martínez, Vicente Adrián Canizalez-Román, Noé Ontiveros, Francisco Cabrera-Chávez

**Affiliations:** 1 Department of Chemical and Biological Sciences, University of Sonora, Hermosillo 83000, Sonora, Mexico; gilberto.aramburo.g@gmail.com; 2 Department of Medicine and Health Sciences, University of Sonora, Hermosillo 83000, Sonora, Mexico; nsotelo51@gmail.com; 3 Division of Sciences and Engineering, Department of Chemical, Biological, and Agricultural Sciences, University of Sonora, Navojoa 85880, Sonora, Mexico; lilian.flores@unison.mx; 4 Laboratory of Immunology and Virology, East Center for Biomedical Research (CIBIOR), Mexican Social Security Institute (IMSS-HGZ No. 5), Metepec 74360, Puebla, Mexico; control37@hotmail.com; 5 Technological Institute of the Yaqui Valley, Bácum 82276, Valle del Yaqui, Sonora, Mexico; mgracia.valenzuela@itvy.edu.mx; 6 Nutrition Sciences Academic Unit, University of Sinaloa, Culiacán 80019, Sinaloa, Mexico; chiquete_90@hotmail.com (F.I.R.C.-E.); jesus.93106@gmail.com (J.G.E.-A.); 7 School of Medicine, University of Sinaloa, Culiacán 80019, Sinaloa, Mexico; adriancanizalez@hotmail.com; 8 Regional Program for Ph.D. in Biotechnology, Faculty of Chemical and Biological Sciences (FC-QB), University of Sinaloa, Culiacán 80030, Sinaloa, Mexico

**Keywords:** food allergy, murine model, sensitization potential

## Abstract

Most food allergy cases are associated with a limited group of allergens. This could be attributed to an increased ability of some foods to sensitize and trigger allergic reactions. However, there are no validated animal models to evaluate the sensitizing or allergenic potentials of proteins. Our aim was to evaluate three protocols of adjuvant-free intraperitoneal sensitization that differ in the time points for sample collection (days 14, 28 and 35 from beginning of the sensitization) and also in the number of immunizations (2, 5 and 3, respectively). Ovalbumin (OVA; 0.05 mg), cow milk proteins (CMP; 0.025, 0.05 and 0.25 mg), and potato acid phosphatase (PAP; low allergenic protein; 250.0 mg) were administered intraperitoneally (ip) to BALB/c mice (n = 4–6) and the protein-specific IgE and IgG antibody responses were evaluated using ELISA. Additional serum protein-specific IgE antibodies evaluations were carried out after IgG depletion. Anti-OVA IgE antibodies were detected in mice from all three protocols. The responses were higher in the group of mice that underwent the 28-day protocol than in those that underwent the 14- or 35-day protocols (*p* < 0.01 and *p* < 0.05, respectively). Anti-CMP IgE antibodies were detected in both the 14- and 28-day protocols, but the response was higher in the group that underwent the 28-day protocol (*p* < 0.001). The anti-CMP IgE antibody response detection was improved after serum IgG depletion (*p* < 0.001). Anti-PAP IgE antibodies were not detected. Mice with undetectable serum levels of protein-specific IgE triggered anti-OVA, -CMP, and -PAP IgG responses. An adjuvant-free 28-day protocol with five ip immunizations seems appropriate for evaluation of the inherent sensitizing or allergenic capacity of the studied proteins. Reproducible results were obtained utilizing the BALB/c mouse strain. Inter-laboratory studies including a larger number of proteins should be carried out to validate this model.

## 1. Introduction

IgE-mediated food allergy is an adverse food reaction triggered by the ingestion of allergenic proteins in sensitized individuals. This disorder affects more than 1%, but less than 10%, of the general population [1]. Sensitization per se is not enough to trigger the symptoms associated with allergic reactions, but is essential for potential IgE-mediated allergic disease. The clinical manifestations of the condition vary from mild and transient symptoms to life-threatening anaphylaxis. Certainly, most cases of food allergy are associated with a limited number of allergens, such as peanut, tree nuts, hen’s egg, cow’s milk, fish and shellfish, although the number of food allergens is large [2,3]. This could be attributed to an increased ability of these foods to sensitize and trigger allergic reactions and suggests that “a spectrum of allergenic or sensitizing potentials exists amongst food proteins” [4]. If such a spectrum does exist, the sensitizing potential of proteins, defined as the inherent capacity of proteins to trigger an IgE-mediated immune response, could be assessed not only in naturally occurring protein allergens, but also in food proteins derived from transgene products or proteins modified upon food processing.

One study suggested that only an in vivo model could be used to evaluate the allergenicity of proteins resulting from de novo sensitization [5], but there are currently no validated animal models available to evaluate the sensitizing or allergenic potential of proteins [6]. A model for the evaluation of such potentials should be able to identify and distinguish commonly allergenic from rarely allergenic proteins [7] by utilizing a standardized adjuvant-free sensitization procedure. Although sensitization to proteins via intraperitoneal (ip) injection has some limitations, it seems to be the method of choice in the mouse model. This is mainly because ip sensitization avoids induction of oral tolerance to administered protein.

The BALB/c mouse strain is considered to favor type 2 immune responses with atopic-like phenotype [8,9], therefore protocols of ip sensitization of BALB/c mice have been proposed [10,11]. Notably, these protocols generate consistent and reproducible results from an intra-protocol point of view. However, the robustness of the IgE antibody response triggered after the ip administration of the reference allergen ovalbumin (OVA; the major allergenic component of egg protein) significantly differ between the proposed BALB/c protocols [11]. In order to validate animal models for the evaluation of the sensitizing potential of proteins, the sensitization protocols should be evaluated using common, weak, and rare allergens. In particular, to evaluate whether the protocol distinguishes commonly allergenic from rarely allergenic proteins. Thus, the aim of this study was to evaluate the use of the BALB/c mouse model to measure sensitizing potential of the common allergens OVA and cow milk protein (CMP), and the rarely allergenic potato protein (PAP) using three different IgE protocols.

## 2. Materials and Methods

### 2.1. Animals

Six- to Eight-week-old female BALB/c mice were purchased from Bioterium Claude Bernard (Benemérita Universidad Autónoma de Puebla, Puebla, México). The mice were maintained on a cow’s milk-egg-potato protein-free standard diet for at least three generations (Mazuri Rat and Mouse Diet #5663) and housed in an animal room at 23 ± 3 °C and 50 ± 10% relative humidity with a 12:12-h light-dark cycle. Water and diet were available ad libitum. The ethics review board of the Autonomous University of Sinaloa (Universidad Autónoma de Sinaloa) approved the study design (Ethical approval number: CE-UACNyG-2014-JUL-001).

### 2.2. Test Materials

The reference allergen OVA (grade V ≥98% pure) and the hypoallergenic potato acid phosphatase (PAP, 0.5–3.0 unit/mg solid) [10,12] were obtained from Sigma Chemical. Cow’s milk protein rich in casein (milk protein) was obtained from MP Biomedicals (Solon, OH, USA) (protein content ≥ 99%, Cat. 0219509605-5). For ip injection, the proteins were solubilized in sterile PBS 7.4 (SIGMA) as follows: (a) 0.02% OVA solution, (b) 10% PAP solution, and (c) 1, 0.5 and 0.1 mg/mL milk protein solutions. Milk protein was macerated until a fine powder was obtained. Samples (5 mg/mL) of milk protein powder in PBS were heated at 50 °C for 1 h with continuous shaking (400 RPM) in a ThermoMixer^®^ C (Eppendorf, Hamburg, Germany). After this, the casein samples were centrifuged at 13,400× *g* for 5 min and the supernatants collected and stored at −80 °C until their use. The protein content of the milk protein preparations was determined using the bicinchoninic acid method according to the manufacturer’s instructions (BCA assay, PierceTM Thermo Scientific, Rockford, IL, USA).

### 2.3. Milk Protein Gel Electrophoresis

The purity analysis of cow milk proteins (MP Biomedicals) was carried out by gel electrophoresis in reducing conditions (SDS-PAGE) according to the method of Laemmli [13]. Commercially available 4–15% polyacrylamide electrophoresis gels were utilized (Mini-PROTEAN^®^TGX Stain-Free, BIO-RAD, Hercules, California, USA). Standard markers (BIO-RAD, Cat. 161-0363) containing 10 proteins ranging in size from 10 to 250 KDa were included on the gels. The cow milk proteins were dissolved in 1X Laemmli buffer (BIO-RAD, Cat. 161-0747) to a final concentration of 1 mg/mL. Samples (20 μL) at different concentrations (2, 4, 6, 8, 10, 12, 14, 16 μg of cow milk proteins) were subjected to SDS-PAGE. Protein bands were visualized with the ChemiDoc Imaging System (BIO-RAD) and analyzed with the Image Lab™ Software version 5.2.1 (BIO-RAD).

### 2.4. Sensitization Procedure

**Figure 1:**
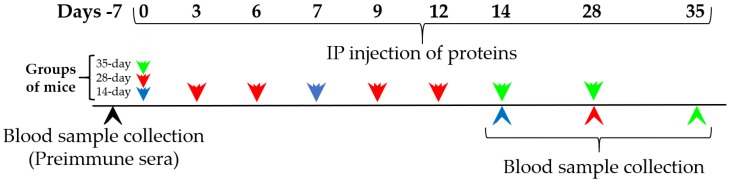
Scheme of sensitization and blood collection. Green, red and blue arrows represent the 35-, 28- and 14-day protocols of sensitization to food proteins respectively.

Groups of mice (n = 4–6) were injected with 250 μL of 0.02% OVA (0.05 mg per mouse), 10% PAP (250.0 mg per mouse) or a range of concentrations of CMP (1, 0.2 and 0.1 mg/mL) in PBS. The control groups (n = 6) were ip injected with 250 μL of PBS only (Sigma-Aldrich cat P5368, Saint Louis, Missouri). The immunization treatments with OVA, PAP and CMP were repeated at different time points: days 0 and 7 for 14-day protocol, days 0, 3, 6, 9 and 12 for 28-day protocol, and days 0, 14 and 28 for 35-day protocol. The mice were exsanguinated (from the tail vein) after 14, 28 or 35 days following exposure to the proteins. The three protocols are described in short in Figure 1. Individual serum samples were stored at −80 °C until analysis.

### 2.5. IgG Depletion of Serum Samples

Serum samples were diluted 1:10 with ELISA diluent (BioLegend. Cat. 408804, San Diego, CA, USA). IgG antibodies were removed from the diluted serum samples. For this purpose, we took advantage of the SureBeads™ Magnetic Beads system (BIO-RAD. Cat. 161-4823). The system contains magnetic beads designed to capture IgG or IgA antibodies. The beats can attach IgG or IgA antibodies from the FC region. The procedure was carried as follows: 100 μL of magnetic beads (SureBeads protein G) were transferred to 1.5 mL tubes. The beads were washed three times with 1 mL of PBS + 0.01% Tween 20. After magnetization of the beads, the wash solution was discarded and 350 μL of diluted serum samples were added into the tube. Samples were left rotating for 10 min at RT and magnetized again. The supernatants (serum samples depleted in IgG) were collected and stored at −80 °C until analysis.

### 2.6. Specific Antibody Analyses

OVA-, PAP- or cow milk protein-specific IgE and IgG were detected using enzyme-linked immunosorbent assay (ELISA). Ninety six-well flat-bottomed polystyrene plates (NUNC Maxisorb #442404) were coated with 20 μg of individual proteins in 100 μL of coating buffer pH 9.5 (BioLegend. Cat. 421701). The plates were incubated overnight at 4 °C, then washed twice with PBS + 0.05% Tween 20 and incubated for 2 h at RT with 200 μL of blocking solution (10% FCS in PBS). The blocking solution was discarded and 100 μL of serum samples diluted 1:10 in ELISA diluent (BioLegend, cat. 421203) were added to individual wells. After overnight incubation at 4 °C, wells were washed three times with PBS + 0.05% Tween 20. 100 μL of the biotinylated detection antibody at 2 μg/mL (Biotin rat IgG1 anti-mouse IgE, BioLegend. Cat. 408804) was added to each well and incubated for 1 h at RT. Wells were washed as before, then 100 μL of streptavidin-horseradish peroxidase (Diluted 1:1000 in ELISA diluent, BioLegend. Cat. 405210) was added to each well and incubated for 30 min at RT. Finally, the assays were developed with 100 μL of tetramethylbenzidine (TMB, Thermo Scientific, Cat. 34028, Rockford, IL, USA) substrate for 30 min at RT. H_2_SO_4_ 2M (50 μL) was used as stop solution. An automated ELISA reader (Multiskan™ FC Microplate Photometer, Thermo Scientific, Cat. 34028, Rockford, IL, USA) was used to measure absorbance at 450 nm. The specific IgG antibodies were determined as stated above, but serum samples were diluted 1:1000 and the biotinylated detection antibody was an anti-mouse IgG at 2 μg/mL (Diluted 1-250 in ELISA diluent, BioLegend. Cat. 405303). The assays were developed for 5 min at RT.

### 2.7. Statistical Analysis

Data were analyzed using GraphPad Prism Version 5.0 (GraphPad Software, San Diego, CA, USA). Normality testing of the data was carried out using Kolmogorov–Smirnov test. Unpaired *t*-tests were used to compare the difference between two different groups and paired *t*-tests were used to compare the levels of antibodies in the same group of animals. One-way ANOVA followed by Tukey’s multiple comparison test for comparison of more than two groups. When normality test failed, Kruskall–Wallis tests followed by Dunn’s multiple comparison tests were used for comparison of more than two groups. Significance was taken to be *p* < 0.05. IgG or IgE antibody responses to antigen were assessed following subtraction of background responses (pre-immune serum samples).

## 3. Results

### 3.1. Electrophoretic Profile of Cow’s Milk Protein

**Figure 2:**
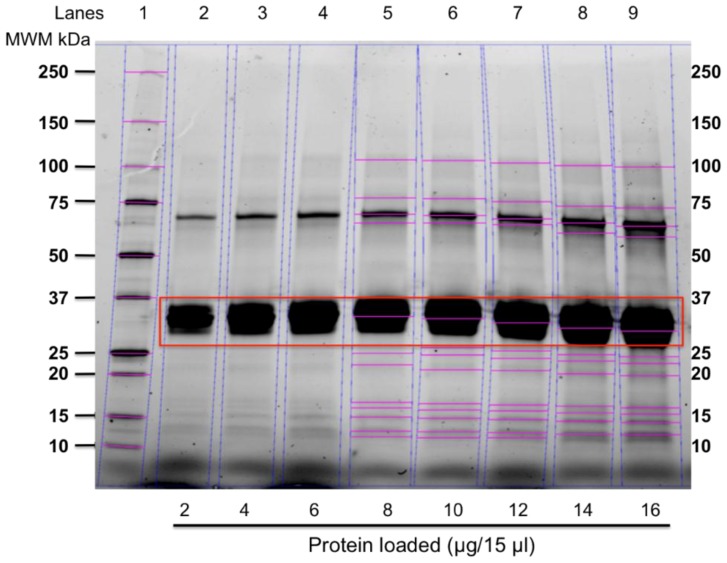
SDS-PAGE of the cow milk protein utilized for sensitization. Lane 1: molecular weight markers (MWM); lanes from 2 to 9: cow milk protein at different concentrations. The lines in the gel show the molecular weight markers (lane 1) and the bands included in the densitometric analysis to determine the percentage of caseins (lanes from 5 to 9). The red box indicates the caseins molecular weight region.

The SDS-PAGE analysis of the cow milk protein revealed the presence of fifteen bands (Figure 2). Caseins and globulins were identified. The percentage of caseins was 87.1%. The densitometric analysis of proteins was carried out considering only the data obtained from lanes 5–9 as at these sample concentrations the image analyzer system detected the total number of bands (Figure 2).

### 3.2. BALB/c Mice Are Efficiently Sensitized to OVA

**Figure 3:**
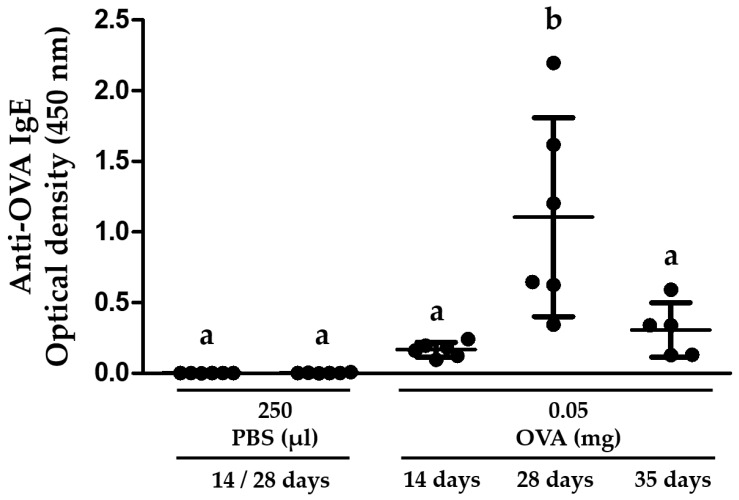
The frequency of ip injections influences the levels of anti-OVA IgE antibodies produced in BALB/c Mice. The animals (n = 5–6 per group) were randomly assigned to a 14-, 28- or 35-day protocol and were injected ip with 250 μL of 0.02% OVA (0.05 mg) for 2, 5, or 3 times respectively (see Figure 1 for details). The mice were exsanguinated after 14, 28 or 35 days according to the protocol used. Different letters mean statistical differences (*p* < 0.05) by one-way ANOVA and Tukey’s multiple comparison tests. All serum samples were evaluated using ELISA.

Three ip protocols of sensitization to OVA were evaluated in this study. The three protocols triggered an anti-OVA IgE immune response that was detected using ELISA (Figure 3). Although the same dose of OVA was administered in each ip protocol, the 28-day protocol with 5 ip injections was more efficient at triggering an anti-OVA IgE immune response than both the 14-day protocol with two injections and the 35-day protocol with 3 injections (*p* < 0.01 and *p* < 0.05, respectively) (Figure 3).

### 3.3. Sensitization to Cow’s Milk Protein Depends on the Dose and Frequency of Ip Injections

**Figure 4:**
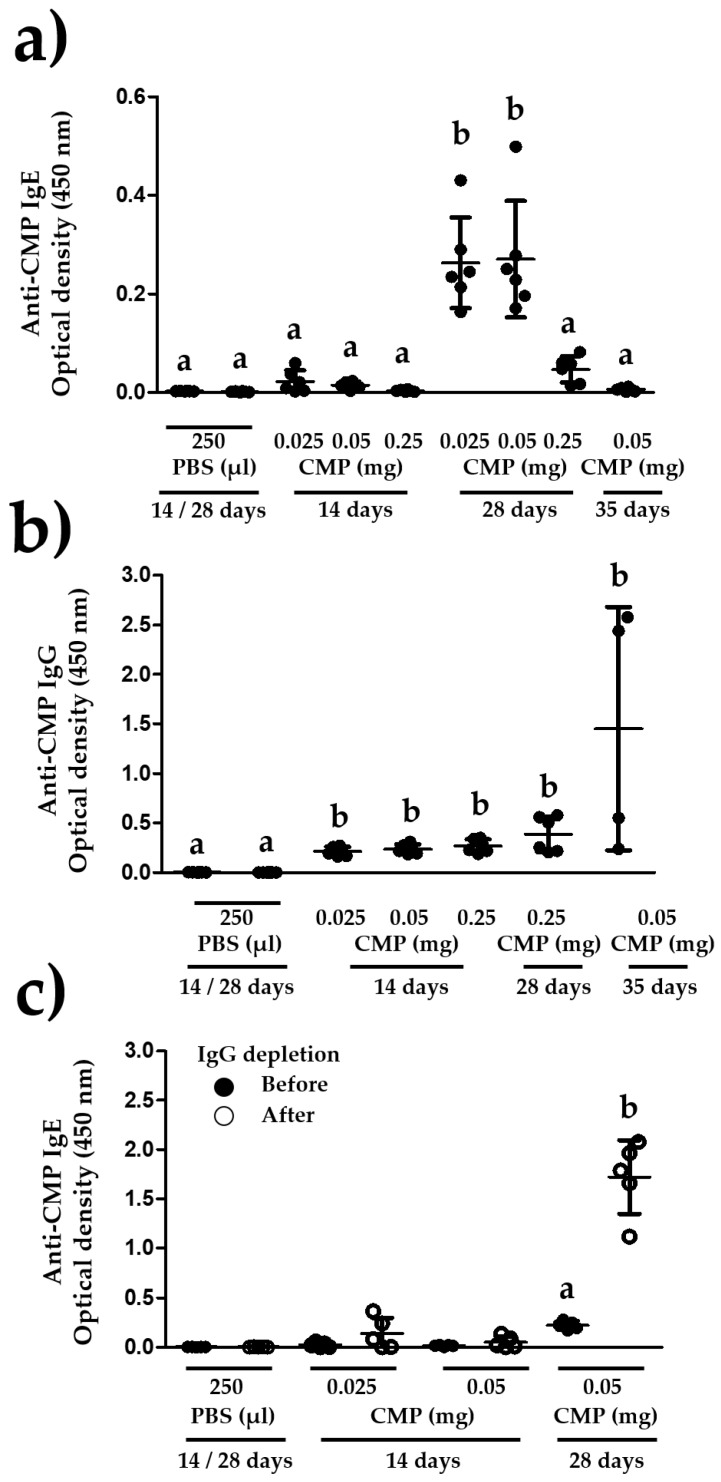
A twenty-eight day protocol with five ip injections efficiently triggers an anti-CMP IgE immune response in BALB/c mice. (**a**) Three protocols of sensitization were evaluated in BALB/c mice (n = 5–6 per group). These protocols differ in the frequency of ip injections and timepoints for blood collection. After the ip injection of different concentrations of CMP (0.025, 0.05 or 0.25 mg) in a final volume of 250 μL, blood samples were collected, and the levels of anti-CMP IgE antibodies evaluated. Different letters mean statistical differences (*p* < 0.05) by one-way ANOVA and Tukey’s multiple comparison tests. (**b**) Serum samples from mice (n = 4–6 per group) with low or undetectable anti-CMP IgE levels were screened for the presence of anti-CMP IgG antibodies. Different letters mean statistical differences (*p* < 0.05) by Kruskall–Wallis test and Dunn’s multiple comparison tests. (**c**) Groups of mice (n = 5) were injected ip with CMP following either a 14- or 28-day protocol with 2 or 5 ip injections respectively. The presence of anti-CMP IgE antibodies in 14- and 28-day serum samples was evaluated before and after IgG depletion. Different letters mean statistical differences (*p* < 0.05) by paired t test. All serum samples were evaluated using ELISA.

Three protocols of adjuvant-free ip sensitization that differ in the number and frequency of injections were evaluated. Two of the protocols were carried out with three different doses of CMP (14- and 28-day protocols). Regarding the 14-day protocol, no anti-CMP IgE antibodies were detected in the sera of mice that were administered 0.25 mg of CMP. The other two groups (0.025 mg and 0.05 mg) showed weak anti-CMP IgE antibodies responses (Figure 4a). The strongest anti-CMP IgE immune responses were triggered after the ip administration of 0.05 mg and 0.025 mg of CMP, and this occurred in the mice from the 28-day protocol. These responses were higher than those triggered by the groups of mice that underwent the 14- or 35-day protocols (*p* < 0.001) (Figure 4a). The 35-day protocol failed to trigger an anti-CMP IgE immune response (Figure 4a). The mice that yielded weak or undetectable anti-CMP IgE antibodies were able to trigger an anti-CMP IgG immune response (Figure 4b). The levels of anti-CMP IgE antibodies were evaluated before and after IgG depletion in three groups of mice. The results showed increased anti-CMP IgE antibodies after IgG depletion, but most serum samples that showed undetectable levels of anti-CMP IgE antibodies remained as such after IgG depletion (Figure 4c). In this same series of experiments, the serum levels of anti-CMP IgE antibodies were significantly increased in a group of mice that underwent the 28-day protocol (*p* < 0.001) (Figure 4c).

### 3.4. A Twenty-Eight Day Protocol with Five Ip Injections Triggers Anti-Pap IgG but Not IgE Antibody Responses in Balb/C Mice

**Figure 5:**
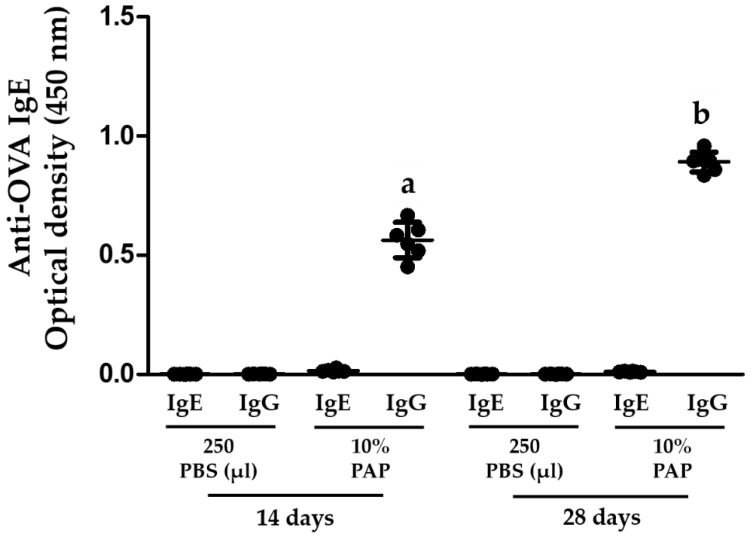
Comparison of the 14- and 28-day protocols to trigger an anti-PAP IgE and IgG antibody response in BALB/c mice. Groups of mice (n = 6) were injected ip with 250 μL of 10% PAP. Blood samples were collected either 14 (2 injections) or 28 (5 injections) days after the first injection. The presence of anti-PAP IgE and IgG antibodies was evaluated using ELISA. Different letters mean statistical differences (*p* < 0.05) by unpaired t test.

Two ip protocols (14- and 28-day) of sensitization were evaluated. As expected, anti-PAP IgE antibodies were not detected in the sera of the mice, after either the 14- or the 28-day protocols, but strong anti-PAP IgG antibody responses were detected in both groups (Figure 5). The IgG antibody responses were higher in the group injected five times (28-day protocol) than in the group that was injected twice (14-day protocol) (*p* < 0.05) (Figure 5).

## 4. Discussion

Mouse models of allergy to a variety of food allergens have been developed [14], but there are no mouse models available to evaluate the inherent sensitizing or allergenic potential of proteins. A model for the evaluation of the previously mentioned potentials requires specific characteristics, such as adjuvant-free conditions, capability to discriminate between commonly allergenic and rarely allergenic proteins, capability to detect allergens that hardly triggers IgE responses under adjuvant-free experimental conditions, and capability to discriminate among common, weak, and rare allergens. Particularly, CMP hardly sensitize mice under adjuvant-free experimental conditions although it is a well-known allergen [15,16]. Contrarily, adjuvant-free protocols to sensitize mice to OVA have been well-documented [11,14,17,18]. In this study, BALB/c mice were used to assess the sensitizing potentials of two commonly allergenic proteins (OVA and CMP) and one rarely allergenic protein (PAP). Two previously published protocols of ip sensitization were followed in detail [8,9,11]. An additional 35-day protocol of ip sensitization was evaluated for comparative purposes, but no anti-CMP IgE immune responses were triggered in the mice that underwent this protocol. These results highlight that the source of allergen is of particular relevance for the evaluation of an animal model for assessing the inherent sensitizing or allergenic potential of proteins.

Previous studies have shown that a 14-day protocol with two ip injections can distinguish commonly allergenic from rarely allergenic proteins [17,18]. However, in these studies the IgE immune response was evaluated by passive cutaneous anaphylaxis assays, an assay that in our hands is technically demanding and difficult to implement. Alternatively, a 28-day protocol to evaluate the allergenic potential of proteins has been proposed [11]. The authors claim that the 28-day protocol triggers more robust IgE immune responses than the 14-day using the reference standard OVA [11] and, notably, the anti-OVA IgE antibodies produced could be readily detected using ELISA. Our results highlight that BALB/c mice that underwent the 28-day protocol with five ip injections and OVA doses of 0.05 mg triggered significantly higher anti-OVA IgE responses than the 14-day protocol with two ip injections. Furthermore, the 28-day protocol not only triggers robust IgE immune responses, but also can discriminate between commonly allergenic and rarely allergenic proteins, such as OVA and PAP respectively. Therefore, our data support the notion that the 28-day protocol is more suitable than 14-day to develop a BALB/c mouse model to evaluate the inherent sensitizing or allergenic potential of proteins.

To corroborate our data with previous findings, the three protocols were carried out utilizing CMP. This commonly allergenic protein was chosen because there is scarce information describing adjuvant-free protocols to sensitize mice to CMP [14]. Previous studies have shown that a six-week-transdermal sensitization protocol is effective to sensitize BALB/c mice to CMP under adjuvant-free conditions [15]. Others have shown that BALB/c mice can be sensitized to CMP without the use of adjuvants after ip injections, but the mice have to be born and grew up in germ-free conditions [16]. The 28-day protocol has the advantages of being shorter than the six-week-transdermal sensitization procedure, and that BALB/c mice can be sensitized after ip injections without the need of germ-free animal facilities. Furthermore, our results show that the 28-day protocol can detect allergens that hardly trigger IgE immune responses under adjuvant-free experimental conditions. Most notably, the IgE responses are robust in a dose dependent manner and are readily detected using ELISA. Overall, the findings highlight a critical role for the dose of allergen, as well as the number and frequency of immunizations, to successfully sensitize BALB/c mice to food proteins, as stated by others [16,19].

Additionally, to show that the undetectable serum levels of anti-CMP IgE antibodies from the 14-day protocol mice were not due to IgG masking of epitopes [20,21], the serum levels of anti-CMP IgE antibodies were evaluated after IgG depletion. After the depletion treatment, anti-CMP IgE antibodies remained undetectable in most serum samples. Contrary, serum samples with detectable levels of anti-CMP IgE antibodies significantly increased the level of detection after IgG depletion showing that IgG masking of epitopes negatively impact on the detection of allergen-specific IgE antibodies.

Animal models have been widely used to evaluate the immune response to antigens in health and disease. The mouse seems to be the animal model of choice to evaluate the inherent sensitizing and allergenic potential of proteins, largely due to the availability of many mouse-specific immunological reagents, their short generation time, the possibility of large experimental groups of animals, and the low cost of purchase and maintenance [22]. Furthermore, it has been reported that the overall structure of the mouse immune system is quite similar to humans [23]. Certainly, the genetic background of each mouse strain has a close association with the type and intensity of the immune response they trigger after antigen exposure, and this characteristic should be taken into account in the search of a mouse model to evaluate the inherent sensitizing or allergenic potential of food proteins [24]. Notably, the BALB/c mouse strain is considered to favor type 2 immune responses with atopic-like phenotype) [8,9,25,26]. Thus, this mouse strain has been widely used in the search for a validated murine model to evaluate the sensitizing or allergenic potential of proteins [4,5,8,9,11].

We should acknowledge that our study has some limitations. Firstly, the anti-IgG immune responses using the 35-day protocol were evaluated in four mice only, and groups of a minimum of five mice have been suggested to evaluate the allergenic potential of food proteins [27]. Furthermore, due to the inter-individual variability in the immune responses, more than six mice should be tested. This consideration should be taken into account in future studies. Secondly, weak allergens were not tested in this study although the results justify their evaluation. Further studies to demonstrate the capability of the 28-day protocol to discriminate between strong and weak allergens are warranted. Thirdly, our data do not support an explanation about the reasons why the 14-day protocol efficiently sensitizes to OVA, but not to CMP. Despite the previous, the results highlight that the 28-day protocol with five ip immunizations triggers a robust IgE immune response that is readily detected using ELISA, can differentiate between commonly allergenic and rarely allergenic proteins, and can detect allergens that hardly trigger IgE immune responses under adjuvant-free experimental conditions. Therefore, the 28-day protocol could be used as an animal model to evaluate the inherent sensitizing or allergenic potential of naturally occurring proteins, biotechnology-derived proteins, or the impact of food processing on the allergenic potential of proteins.

## 5. Conclusions

An adjuvant-free 28-day ip sensitization protocol with five immunizations and doses of 0.05 mg of OVA or CMP is suitable to trigger an IgE immune response detectable by ELISA in BALB/c mice. Notably, the protocol failed to trigger IgE immune responses, but not IgG, to rarely allergenic proteins such as PAP. Thus, the adjuvant-free 28-day protocol could be a useful tool to evaluate the inherent sensitizing or allergenic potential of proteins in BALB/c mice. Inter-laboratory studies with a larger number of common, weak, and rare allergens should be carried out to validate this model.
